# A cross-sectional study on non-infectious dysfunction of hemodialysis cuffed catheter

**DOI:** 10.22088/cjim.15.3.439

**Published:** 2024-08-01

**Authors:** Pouya Tayebi, Kosar Hasanzadeh, Masoumeh Asgharpour, Ali Bijani, Naghmeh Ziaie

**Affiliations:** 1 Department of Vascular and Endovascular Surgery, Ayatollah Rouhani Hospital, Babol University of Medical Sciences, Babol, Iran; 2 Student Research Committee, Babol University of Medical Sciences, Babol, Iran; 3 Department of Nephrology, Ayatollah Rouhani Hospital, Babol University of Medical Sciences, Babol, Iran; 4 Social Determinant of Health Research Center, Babol University of Medical Sciences, Babol, Iran; 5 Department of Cardiology, Ayatollah Rouhani Hospital, Babol University of Medical Sciences, Babol, Iran

**Keywords:** Renal dialysis, vascular catheters, Thrombosis, Fibrin adhesive

## Abstract

**Background:**

Dialysis cuffed catheter dysfunction results in inadequate dialysis, increased sepsis risk, and a shortened catheter life. It may be possible to prolong catheter function by identifying the causes of cuffed catheter dysfunction.

**Methods:**

This study was a cross-sectional descriptive study conducted in 2021-2022 on hemodialysis patients with jugular cuff catheters. The catheterizations were performed using the Seldinger technique and were confirmed by fluoroscopy. A 12-month follow-up was conducted with respect to the performance of the cuffed catheter.

**Results:**

A total of 123 patients underwent hemodialysis over 2 years via a cuffed catheter. Catheters were most commonly inserted into the right internal jugular vein, with lengths of 19 cm (tip to cuff). The rate of dysfunction of cuffed catheters was 27.6%. Catheter-related thrombosis was the most common cause in 10 cases (29.4%), followed by catheter tip fibrin sheath in 8 cases (23.5%) and catheter tip malposition in 8 cases (23.5%). Furthermore, 18 patients (52.94%) of cuffed catheter dysfunction occurred within 3 months of catheter placement, based on our study. The dysfunction of cuffed catheters on the left side 23 (67.64%) is more prevalent than the right side 11 (32.35%) (P=0.043); the malposition of catheter tips is more prevalent on the left side (P=0.023).

**Conclusion:**

Most commonly, cuffed catheter dysfunction is caused by thrombosis, fibrin sheath formation, and catheter tip malposition. Cuffed catheter failure can be reduced by carefully monitoring the catheter's path and tip position, searching for fibrin sheaths when investigating cuffed catheter failure, and preventing thrombotic events.

A tunneled central venous cuffed catheter is typically less effective than an arteriovenous fistula (AVF) or graft (AVG) (1). It is typically used by patients who lack access to dialysis permanently or are unable to obtain an AVF or AVG suitable for dialysis, yet do not have a permanent access to dialysis (2). While these catheters provide temporary vascular access for hemodialysis, they are associated with several risk factors for dysfunction, which can lead to complications such as infection, thrombosis, and inadequate dialysis (3). The risk factors for cuffed hemodialysis catheter dysfunction are multifaceted, encompassing catheter-related factors, patient-related factors, care and maintenance practices, catheter duration, inflammation, patient education, and healthcare provider expertise. The incidence of catheter-related bloodstream infections is reported to be 9%, catheter dysfunction 15%, and central vein stenosis 2%, whereas deaths associated with central venous catheters occur in only 0.5% of patients (4). In published articles, within six and twelve months following tunneled central vein catheter implantation, the rate of dysfunction is between 35% to 47% (5-7) and to 15% to 66% (7-9) respectively. 

A catheter can be considered dysfunctional when it is unable to do two consecutive dialysis periods without causing recurrent pressure alarms or when it cannot produce hand over blood flow levels exceeding 300 ml/min on two consecutive dialysis sessions (3, 10). There are several causes of catheter dysfunction, including thrombosis, fibrin sheath obstruction, and mechanical problems. Also, central vein obstruction can be detected in patients with > 70% stenosis of a central vein, or upper limb edema on the same side, and history of central vein catheterization (3). Considering the high rate of cuffed catheters dysfunction in one year after implantation, it is necessary to identify the underlying causes and fix them.

## Methods

A cross-sectional descriptive study conducted at Ayatollah Rouhani Hospital in Babol city, North of Iran in 2021-2022, looked at patients with advanced chronic renal failure who received hemodialysis three times a week through jugular cuffed catheters for four hours each time. The inclusion criteria were defined as applicants must be between 20 and 80 years old, be dialyzed at least once a week using a jugular cuffed catheter, have no venous obstruction at the location of the cuffed catheter, and do not have a history of venoplasty in upper central veins. Those who started antiplatelet or anticoagulant treatment or had a fistula or graft on the same side as the cuffed catheter were excluded from the study. Catheterizations were performed using ultrasound guidance (Seldinger technique) and confirmed with fluoroscopy as the catheter was properly inserted into the right atrium. There was a 12-month follow-up on the performance of the cuffed catheter. When a catheter cannot be used for two consecutive dialysis sessions, it is considered dysfunctional and in order to investigate the cause of cuffed catheter dysfunction, venography of the upper central veins was performed through the catheter itself. Thrombosis, fibrin sheath obstruction, catheter route, and central vein obstruction were investigated as causes of catheter dysfunction. Data was analyzed using SPSS Version 25 statistical software. The variables were described using the average standard deviation for quantitative variables and frequency and percentage for qualitative variables. t-test, chi-square and regression tests were applied to analyze the data and p<0.05 was considered significant. This study has been approved by the Ethics Committee of Babol University of Medical Sciences (IR.MUBABOL.REC.1399.504).

## Results

123 patients, including 50 (40.7%) men and 73 (59.3%) women, with an average age of 61.55 ± 11.61, underwent hemodialysis through a cuffed catheter during 2 years. Hypertension 107 (87%), diabetes mellitus 97 (78.9%) and hyperlipidemia 62 (50.4%) were the most common comorbidities among the studied subjects. Among the patients included in the study, catheterization characteristics were as follows: catheter length (from tip to cuff) was 19 cm for 92 (74.79%) patients and 23 cm for 31 (25.20%) patients. The site of catheter insertion was predominantly the right internal jugular vein, utilized by 98 (79.67%) patients. A smaller proportion of patients had catheters inserted in the right subclavian vein (20 patients, 16.26%), the left internal jugular vein (4 patients, 3.25%), and the left subclavian vein (1 patient, 0.81%).

**Table 1:** Cuff catheter dysfunction etiologies in studied patients

**Cause of catheter dysfunction**	**Number (%)**	**Cuff catheter insertion site**		
		**Right**	**Left**	**P-value**
Catheter-related thrombosis	10 (29.4%)	4 (11.76%)	6 (17.67%)	0.091
Catheter tip fibrin sheath	8 (23.5%)	3 (8.82%)	5 (14.70%)	0.078
Catheter tip malposition	8 (23.5%)	1 (2.94%)	7 (20.58%)	0.023
Central venous stenosis	3 (8.8%)	1 (2.94%)	2 (5.88%)	0.082
Incorrect length of catheter	2 (5.9%)	1 (2.94%)	1 (2.94%)	0.062
Catheter kink	2 (5.9%)	1 (2.94%)	1 (2.94%)	0.076
Incorrect location for venipuncture	1 (2.9%)	0	1 (2.94%)	0.069
Total	34 (100%)	11 (32.35%)	23 (67.64%)	0.043

The rate of cuffed catheter dysfunction was (34 27.6%) people. In table 1, all the causes of dysfunction of cuffed catheters are listed. The most common cause of cuffed catheter dysfunction was catheter-related thrombosis in 10 (29.4%) cases, followed by catheter tip fibrin sheath in 8 (23.5%) cases and catheter tip malposition in 8 (23.5%) cases (table 1). Our findings show that the dysfunction of cuffed catheters on the left side 23 (67.64%) is more than the right side 11 (32.35%) (P=0.043) specially the malposition of catheter tips on the left side is more prevalent than on the right side (P=0.023), among the existing causes (table 1). Also, based on the results of our study 18 (52.94%) patients of cuffed catheter dysfunction occurred during the first 3 months after catheter insertion, and 11 (32.35%) patients occurred during the following 9 months (table 2)

**Table 2:** Cuff catheter dysfunction in studied patient during 1 year follow up

**Time of catheter dysfunction**	**Number (%)**	**Cause of dysfunction**	**Number (%)**
**< 3 months**	18 (52.94%)	Catheter-related thrombosis	5 (14.70%)
		Catheter tip fibrin sheath	1 (2.94%)
		Catheter tip malposition	7 (20.58%)
		Incorrect length of catheter	2 (5.88%)
		Catheter kink	2 (5.88%)
		Incorrect location for venipuncture	1 (2.94%)
**3-6 months**	5 (14.70%)	Catheter-related thrombosis	2 (5.88%)
		Catheter tip fibrin sheath	2 (5.88%)
		Catheter tip malposition	1 (2.94%)
**6-12 months**	6 (17.64%)	Catheter-related thrombosis	2 (5.88%)
		Catheter tip fibrin sheath	3 (8.82%)
		Central venous stenosis	1 (2.94%)
**>12 months**	5 (14.70%)	Catheter-related thrombosis	1 (2.94%)
		Catheter tip fibrin sheath	2 (5.88%)
		Central venous stenosis	2 (5.88%)

## Discussion

Catheter-related thrombosis, fibrin sheath formation, and catheter tip malposition were the leading causes of cuffed catheter dysfunction in our study. Early catheter dysfunction is generally caused by mechanical issues, such as patient malpositioning, mechanical kinking of the catheter and incorrect catheter tip placement (11, 12). The catheter tip should be placed at the level of the mid-right atrium (13) , and fluoroscopy should be used to confirm this position. For catheters placed on the left side, this may be more important than for catheters placed on the right side (14). It is also consistent with our study results that the overall rate of left cuffed catheter dysfunction is higher than that of the right side. In addition, the improper location of the catheter tip is significantly more prevalent in left side cuffed catheters. Moreover, it was found that catheter-related thrombosis was the most common cause of cuffed catheter dysfunction in our study (29.4%). As observed in the Sahli’s study (15), a similar percentage was observed in 46.7% of cases with hemodialysis catheters. Based on the results of a study carried out in Croatia (16) among dialysis patients who use central catheters for dialysis, this rate was 16.8 percent. As per Develter et al., (17) thrombosis was the cause of 12% of catheter malfunctions, which is significantly lower than the findings of our study. Multiple factors can explain this difference in thrombotic causes, including underlying diseases, drugs, and hemodynamic conditions. Fibrin sheath formation I another cause of catheter dysfunction. According to a previous study, fibrin sheath formation produces catheter dysfunction in 13% to 57% of hemodialysis patients (18), which is similar to our study's 23.5%. Although the technical causes of dysfunction of cuffed catheters were few in our study, tunneling and catheter insertion technique plays an important role. A technical error could lead to the catheter kinking and malfunctioning, preventing the patient from being able to perform hemodialysis as a result. The importance of x-ray control of the catheter position just after or during the placement should never be underestimated, regardless of what situation may arise (19). 

As a result, it could reduce the incidence of delayed detection of catheter malpositions in the future. It has been shown that the absence of such confirmation will result in a delayed catheter reposition, which will increase the risk of infection later during manipulating the catheter. Kinking occurred in 5.9% of our patients as a result of technical error. Hamid et al. (20), Gobleowski et al. (19), and Wong et al. (21) reported similar failure rates caused by catheter kinking or pinching in 4.9%, 2.7%, and 0.6% of all catheter placement procedures. It is necessary in such situations to exchange the line and additional interventions may be required. In conclusion, catheter-related thrombosis, fibrin sheath formation, and catheter tip malposition constitute the most common causes of cuffed catheter dysfunction. Therefore, it is possible to reduce the initial failure rate of cuffed catheters by carefully monitoring the catheter path and the position of its tip, searching for fibrin sheaths when investigating the causes of cuffed catheter dysfunction, and preventing thrombotic events if possible.
